# The Young and Sexual Hygiene

**Published:** 1912-09

**Authors:** 


### The Young and Sexual Hygiene

The question of the necessity for education of the young in sexual hygiene has absolutely forced itself upon the attention of the community. For years it has been well known that there was crying need for such education, but owing to the prudery, inbred and instinctive, of the so-called Anglo-Saxon race, the matter has been blinked at and shelved. Of recent years, however, a new spirit appears to be animating the English-speaking peoples in this respect, and now it is generally allowed that it is strongly advisable to instruct the young in sexual hygiene, and that the subject should no longer be taboo. Also, it is to be noted, even the conservative British physicians are taking up the matter and arguing that the time is ripe for theories in this direction to be put into practice. Dr. Eric Pritchard writes in the *Medical Press and Circular,* June 5, 1912, in favor of the instruction of the young in sexual hygiene. He points out that the reason why children should be taught sexual morality is connected partly with their own individual interests, and partly with the wider interests of race preservation. For the child's own sake it is desirable that it should have an intelligent comprehension of the physiological processes of life, including that of reproduction, in order that at the time of puberty it may understand the significance of those radical changes which herald its physico-sexual maturity. Such knowledge is necessary in order that the psychological shock of a too rude awakening may be avoided at this crisis in the child's life, and in order that it may be armed at a somewhat later period against the consequences of venereal diseases, and of physical and moral corruption.

With regard to a most important point, that of the best time to begin sexual instruction, Pritchard thinks that it would be advisable to confine our energies to the instruction of children who are about to leave school and are going out into the world with all its dangers and temptations. Possibly British children leave school at an earlier age than they do in this country, or else they must be much more innocent than American children. Here, it is safe to say, school children who do not know the broad facts of sex life and reproduction long before their school life is ended are few indeed, and Pritchard would be too late with his instruction; he would find the field already grown up in weeds with little room for the seeds of morality he might sow to take root. We do not mean to say all children of this age are corrupt—indeed, few are, but if those few are to be saved by knowledge their instruction must be begun at an early age; at the end of school life is too late. More emphatic is his opinion that it is of equal importance that teachers and parents should likewise be taught many of the strange manifestations of the sexual instinct of which at the present time they are in complete ignorance. They do not know what to expect, what to anticipate, or how to recognize the symptoms when they appear, and are consequently ill-adapted to train the children under their direction. In conclusion, Pritchard suggests that we should all make an effort to face the difficulties of the situation and free ourselves of that shame-faced self-consciousness which has in the past enveiled the whole question with an atmosphere of unnatural and artificial indecency—indecency which does not exist except in the imagination. Teachers, parents, and physicians should divest themselves of this false conception and attempt to arrive at some satisfactory solution of the problem.—Editorial, *New York Medical Record*, July 20th.

